# Plant Disease Resistance-Related Signaling Pathways: Recent Progress and Future Prospects

**DOI:** 10.3390/ijms232416200

**Published:** 2022-12-19

**Authors:** Li-Na Ding, Yue-Tao Li, Yuan-Zhen Wu, Teng Li, Rui Geng, Jun Cao, Wei Zhang, Xiao-Li Tan

**Affiliations:** College of Life Sciences, Jiangsu University, Zhenjiang 212013, China

**Keywords:** disease resistance, signal molecule, plant hormone, signaling pathway

## Abstract

Plant–pathogen interactions induce a signal transmission series that stimulates the plant’s host defense system against pathogens and this, in turn, leads to disease resistance responses. Plant innate immunity mainly includes two lines of the defense system, called pathogen-associated molecular pattern-triggered immunity (PTI) and effector-triggered immunity (ETI). There is extensive signal exchange and recognition in the process of triggering the plant immune signaling network. Plant messenger signaling molecules, such as calcium ions, reactive oxygen species, and nitric oxide, and plant hormone signaling molecules, such as salicylic acid, jasmonic acid, and ethylene, play key roles in inducing plant defense responses. In addition, heterotrimeric G proteins, the mitogen-activated protein kinase cascade, and non-coding RNAs (ncRNAs) play important roles in regulating disease resistance and the defense signal transduction network. This paper summarizes the status and progress in plant disease resistance and disease resistance signal transduction pathway research in recent years; discusses the complexities of, and interactions among, defense signal pathways; and forecasts future research prospects to provide new ideas for the prevention and control of plant diseases.

## 1. Introduction

Plants have established multi-level passive and active resistance mechanisms during the course of evolution that can be used coordinately against pathogen infection. Passive resistance mainly involves physical barriers on the plant cell surface and substances inside cells that are toxic to pathogens, such as antibacterial compounds, phenols, unsaturated lactones, and antimicrobial peptides [1,2,3,4,5]. Active defense is mainly induced rapidly after pathogen infection, and it includes the release of reactive oxygen species (ROS), the production of the hypersensitive response (HR), the formation of phytoalexin, and the reinforcement and repair of the cell wall [2,6]. The activation time and intensity of the defense response determine the plant’s resistance level [7].

**Figure 1:**
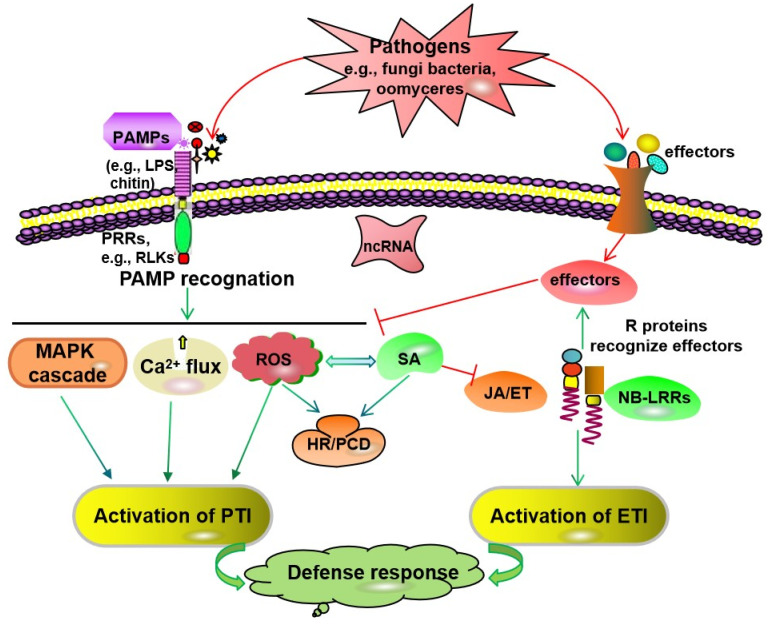
A model of immune responses in plant–pathogen interactions. A plant’s innate immune system consists of PTI and ETI. PTI induced through the recognition of PAMPs by PRRs can inhibit the growth of most pathogens. Then, downstream signaling pathways such as Ca^2+^ signaling, the MAPK cascade, and ROS signaling are activated. Some pathogens can release pathogenic effectors to interfere with PTI, causing susceptibility triggered by effectors. The disease R proteins with conserved NB-LRR can directly or indirectly identify specific effectors to trigger ETI, which often causes an HR at the infection site of the pathogen, and then inhibit the growth of the pathogens again. The SA and JA/ET signaling pathways are also involved in PTI and ETI activation and the resistance response to pathogen infections, thereby stimulating downstream transcription factors and initiating plant defense responses. Many ncRNAs play critical roles in PTI or ETI responses by regulating various biological processes, such as the MAPK cascade, the expression of signaling components, ROS production, plant hormone biosynthesis, and signaling. NB-LRR, nucleotide-binding leucine-rich repeat; PRRs, pattern recognition receptors; SA, salicylic acid; JA/ET, jasmonic acid/ethylene; HR, hypersensitive response; PCD, programmed cell death; SAR, systemic acquired resistance; R, resistance; ROS, reactive oxygen species; MAPK, mitogen-activated protein kinase; PAMPs, pathogen-associated molecular pattern; PTI, PAMP-triggered immunity; ETI, effector-triggered immunity; ncRNAs, non-coding RNAs. The arrows indicate positive regulation, and open blocks indicate negative regulation.

**Figure 2:**
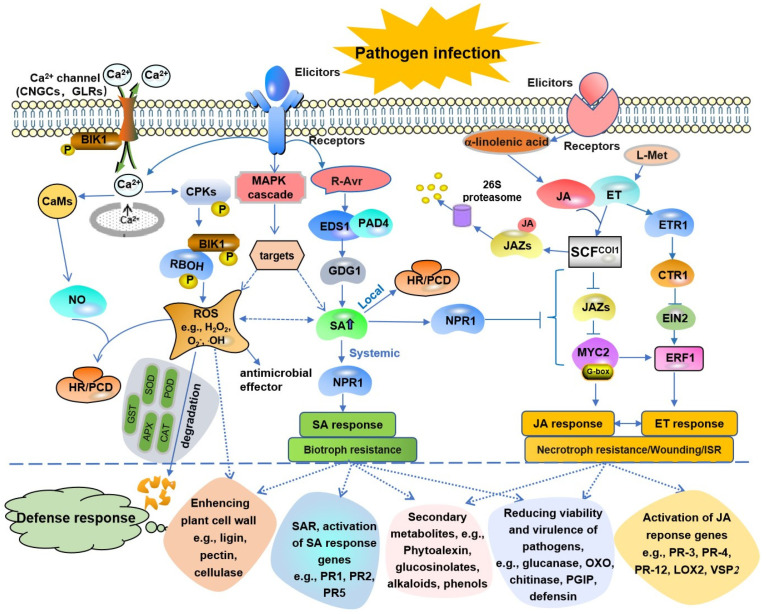
Schematic representation of the plant defense signal transduction network. In the process of plant–pathogen interactions, a series of signals are triggered to induce plant defense responses. The complex and diverse signal pathways interact with each other and form signal transduction networks in plants. After the resistant host recognizes the elicitors produced by pathogens, it activates the signal transduction system, causing the release of Ca^2+^, an MAPK cascade reaction, and the activation of R genes. Ca^2+^ flowing into the cytoplasm can activate CaMs and CMLs, induce downstream NO synthesis, and then induce a primary immune response, including HR. Moreover, NO may regulate an HR/PCD through a synergistic effect with ROS. Besides being a signal to activate SAR, ROS can directly act as antibacterial effectors and enhance the structural resistance of the host. The interaction between the *R* gene and the avirulent gene (*Avr*) can stimulate a series of defense responses, such as the HR/PCD and SAR, which are induced by SA and give a strong resistance against some biotrophic pathogens. *GDG1* is necessary for pathogen-induced SA biosynthesis, and its expression is regulated by *EDS1* and *PAD4*. SA can also control the expression of *GDG1*, *EDS1*, and *PAD4* through a positive feedback loop. The positive feedback regulation of *GDG1* lies in the high level of SA accumulation. JA/ET signaling pathways mainly regulate plant resistance to necrotic pathogens and wounds. JA and ET also mediate the resistance induced by root microorganisms, which is called induced systemic resistance (ISR). SA inhibits the JA/ET pathway by activating *NPRl*, a positive regulatory gene of the SA pathway. ERF1 is located at the intersection of the JA and ET signaling pathway defense against pathogen infections and in wound responses. A JA signal can promote the interaction between JAZ and the SCF^COI1^ ubiquitin ligase, resulting in the ubiquitination of the JAZ protein and degradation by the 26S proteasome, and then the activation of transcription factors such as MYC2 to induce JA responses. Ultimately, a series of downstream responses, such as the reinforcement of physical defensive structures, the production of secondary metabolites, the inhibition of growth pathogens by the induction of defensive proteins, the activation of the ROS scavenging system, and other disease resistance factors, are activated to fight against pathogen infection. CNGCs, cyclic nucleotide-gated channels; CaMs, calmodulins; CPKs, calcium-dependent protein kinases; GLR, glutamate receptor-like genes; NO, nitric oxide; POD, peroxidase; SOD, superoxide dismutase; CAT, catalase; APX, ascorbate peroxidase; GST, glutathione S-transferase; ROS, reactive oxygen species; RBOHD, respiratory burst oxidase homolog D; H_2_O_2_, hydrogen peroxide; O₂^−^, superoxide ion; OH, hydroxyl radical; BIK1, BOTRYTIS-INDUCED KINASE1; MAPK, mitogen-activated protein kinase; *R*-*Avr*, interaction between an avirulence (*Avr*) gene in the pathogen and the corresponding resistance (*R*) gene in the host; EDS1, enhanced disease susceptibility; PAD4, phytoalexin-deficient 4; NPR1, non-expresser of PR genes 1; GDG1, GH3-like defense gene 1; EIN2, ethylene-insensitive 2; ISR, induced systemic resistance; SCF, Skp/Cullin/F-box; COI1, coronatine-insensitive 1; LOX2, lipoxygenase 2; VSP2, vegetative storage protein 2; JAZ, jasmonate ZIM-domain; CTR1, copper transport protein 1; ETR1, ethylene receptor gene 1; ERF, ethylene response factor; PR, pathogenesis-related protein; PGIP, polygalacturonase-inhibitory protein; OXO, oxalate oxidase. The arrows indicate positive regulation, and open blocks indicate negative regulation. Dashed lines indicate possible or indirect interactions.

After plants are infected by pathogens, such as bacteria, fungi, oomycetes, mycoplasma, or viruses, the activation of the defense response is finely regulated. At the infection site, the plant defense response is initiated by two types of molecules that are derived from pathogens. A pathogen-associated molecular pattern (PAMP), recognized by cell-surface-localized pattern-recognition receptor (PRR)-triggered immunity (PTI), initiates the first defense line of host-induced defense responses, giving plants a basic resistance to most pathogens. In the resistant host–pathogenic microbe pathosystem, after recognizing the PAMPs produced by pathogens, resistant hosts directly induce PTI, such as calcium ion (Ca^2+^) influx, ROS production, and mitogen-activated protein kinase (MAPK) activation. Then, the downstream salicylic acid (SA) or jasmonic acid (JA)/ethylene (ET) signal pathway is activated, which leads to the biosynthesis of defense-related factors and the activation of disease resistance in plants (Figure 1). The other type of initiator is effector proteins, such as AvrPto, AvrPtoB, and AvrPphB, which are transported to plant cells mostly by the pathogen type III secretion system [8,9,10]. Effector-triggered immunity (ETI) induced by the interactions of plant resistance (R) proteins and pathogen effectors can start the second line of host-induced defense responses (Figure 1). It stimulates a series of defense responses, such as HR/programmed cell death (PCD) and systemic acquired resistance (SAR). These reactions can be induced by SA, resulting in a strong resistance to some biotrophic or hemi-biotrophic pathogens [11]. The JA/ET-mediated pathway is involved in inducing resistance to necrotrophic pathogens [12]. To survive pathogen invasion, plants use a variety of signaling pathways to activate their resistance responses [13]. Therefore, plant-induced resistance is regulated by a complex signal transduction network and involves the expression of a series of resistance-related genes (Figure 2).

At present, it is clear that some signal molecules, such as Ca^2+^, ROS, nitric oxide (NO), the heterotrimeric G protein, and non-coding RNAs (ncRNAs), play important roles in regulating disease resistance and the defense signal transduction network. Both PTI and ETI are regulated by plant hormones, with SA, JA, and ET being the main signals. In addition, the disease resistance response by plants also involves the immune response mediated by MAPK and the subsequent antibacterial products, ROS, defensins, and phytoalexins, which can also enhance the defensive capability against pathogens [14,15]. In recent years, important progress has been made in the research on plant disease resistance signaling pathways. This paper summarizes the progress and forecasts future research to provide a theoretical basis for the prevention and control of plant diseases.

## 2. Plant Messenger Signal Molecule-Mediated Signaling Pathways

### 2.1. Calcium-Mediated Disease Resistance Pathway

Ca^2+^ is a conserved second messenger and the main mediator of plant immune and stress responses. A recent study showed that NRG1.1, a plant nucleotide-binding leucine-rich repeat receptor function in ETI, actually encodes a Ca^2+^-permeable channel [16]. As a ubiquitous signal molecule, Ca^2+^ controls a wide range of cellular metabolic processes, including the regulation of oxidative burst, gene expression, and signal transduction, and it also regulates many key points in the apoptotic process. It is crucial to the regulation of various stress genes involved in plant resistance [17]. The distribution of Ca^2+^ in plant cells is extremely unbalanced. A change in the intracellular free Ca^2+^ concentration may be mainly realized by the transmembrane transport of Ca^2+^ or the regulation of Ca chelates [18]. The Ca^2+^ concentration is crucial for immunity triggered by Ca^2+^-dependent PAMPs in plants. At a sufficient external Ca^2+^ concentration, the encoded cyclic nucleotide-gated channel is the key determinant of Ca^2+^ signaling and PTI responses induced by PAMPs and ROS. After pathogen invasion, the channel is phosphorylated and activated by the effector kinase Botrytis-induced kinase 1 of the pattern-recognition receptor complex, triggering an increase in the intracellular Ca^2+^ concentration [19]. Plant perception of pathogen-related stress usually leads to stomatal closure. The *Arabidopsis thaliana* Ca^2+^ osmotic channel OSCA1.3 and its phosphorylation by Botrytis-induced kinase 1 can control stomatal closure during immune signal transduction [20]. Ca^2+^ signals are also involved in the regulation of biotic/abiotic stress-induced PCD in plants, and Ca^2+^ participation in early apoptosis is particularly important [21].

Intracellular Ca^2+^ signals are transduced through downstream receptor proteins. At present, there are mainly two kinds of calcium receptor proteins in plants: calmodulins (CaMs) and Ca^2+^-dependent protein kinase (CDPK) [21,22]. In *Arabidopsis*, CaMs and calmodulin-like proteins (CMLs) constitute a large Ca^2+^-sensing receptor protein family that translates and transmits Ca^2+^ signals in many signal transduction cascade reactions [23]. In addition, a CaM antagonist can inhibit the PAMP-induced Ca^2+^ channel and nitric oxide synthase (NOS)-mediated induction of NO, suggesting that Ca^2+^, CaMs, and NOS are involved in the signal transduction cascade of plant pathogens [24,25]. Moreover, the CaM transcription activator factor is involved in SA biosynthesis and SA-mediated immune responses [26]. Recent studies have shown that CML proteins, such as CML13 and CML8, are involved in plant defense responses to many pathogens, such as *Pseudomonas syringae* and *Ralstonia solanacearum*, and their overexpression regulates pathogenesis-related (*PR*) genes as well as many genes involved in signal transduction and stress responses [27,28,29]. In recent years, the evidence for CDPKs being involved in plant defense responses has gradually increased. The *A. thaliana CPK5* enhances SA-mediated resistance to the bacterial pathogen *P. syringae* pv. *tomato* strain DC3000, resulting in the differential expression of plant defense genes and the synthesis of ROS [30,31]. Rice *OsCPK10* interferes with the growth of necrotic fungi by reducing the accumulation of hydrogen peroxide (H_2_O_2_), which improves basic disease resistance [32]. However, the overexpression of some CDPKs, such as *AcoCPK1* and *GhCDPK28–6*, weakens plant resistance to necrotic pathogens and reduces hormone response gene expression [33,34]. In short, Ca^2+^, as a very important intracellular signal transduction factor, is crucial to the regulation of plant defense response gene expression and plays a key role in plant innate immunity. However, the specific mechanisms of Ca^2+^ regulation at the molecular level are still unclear.

### 2.2. ROS-Mediated Disease Resistance Signaling Pathway

During biotic or abiotic stress, ROS in plants will be produced rapidly and briefly, resulting in the cellular ROS concentration being significantly higher than normal. This represents the frequently mentioned ‘oxidative burst’ [35,36,37,38]. In addition, glucan, galactosaldehyde, peptides, and SA from pathogenic fungi and their plant hosts can also induce the plants to produce ROS and increase cell mortality [39]. There are five types of ROS, with H_2_O_2_ being a relatively stable type that can diffuse into subcellular intervals, making it a more physiologically important ROS type [40,41]. The plant NADPH oxidase RBOHD is the main participant in ROS production during innate immunity, and the ROS oxidative burst is likely caused by NADPH oxidase activity [42,43]. The C-terminal of RBOHD is regulated by the phosphorylation and ubiquitination of various kinases to ensure the production of a full elicitor-induced ROS burst [44]. The mutation of a phosphorylation site weakens the defense responses of plants against pathogen infection [45]. However, how the ROS level is precisely regulated to avoid cell damage owing to excessive ROS production, and how pathogens tolerate ROS stress, remains unclear.

ROS have dual functions under stress conditions [46]. On the one hand, due to their strong oxidative properties, ROS disrupt the normal metabolism of organisms and also cause the degradation of macromolecular substances, resulting in cell damage, the loss of normal physiological functions, and even death. On the other hand, ROS, as important signal molecules, are active in many biological systems [37,47]. For example, ROS may be used as antibacterial agents and have direct toxicity against invasive pathogens [48]. ROS are also involved in the lignification of the cell wall and the cross-linking of related proteins with the cell wall, strengthening the cell wall and enhancing the structural disease resistance of the host [49,50].

An ROS burst is also considered to be a characteristic response of an HR and one of the earliest responses to pathogens. From the beginning of pathogen infection to the production of plant SAR, a continuous signal transmission process occurs. As a signal in this process, ROS also function as excitation signals to induce the formation of SAR [51,52]. At this point, ROS play dual roles in plant defense responses; that is, at high concentrations, ROS damage plants, but at low concentrations, ROS signal transduction induces the expression of plant resistance and defense genes [53,54]. Although ROS waves have been shown to be essential for rapid systemic signaling, they do not convey the specificity of all systemic responses. Recent studies have shown that ROS waves and calcium waves interact with each other and have potential co-amplification effects [55]. Moreover, ROS, calcium, and electric signals have also been proposed to have a potential link in systemic signal transduction from local tissues to the whole plant [46,56,57]. How these rapid systemic signals interact with and integrate each other under different stresses remains largely unknown.

### 2.3. NO-Mediated Disease Resistance Signaling Pathway

NO is a redox signaling molecule that is widely distributed in organisms and that participates in a variety of physiological processes through the regulation of different post-translational modifications (PTMs) [58,59]. The bioactivity of NO can be initiated by S-nitrosation, a process involving the addition of the NO moiety to protein cysteine thiols to form S-nitrosothiols, which are considered to be important PTMs that control numerous cellular processes associated with plant immunity. In plants, NO can regulate SUMOylation through the S-nitrosation of the SUMO (small ubiquitin-like modifier)-conjugating enzyme, which has been proposed to control the activation of plant immunity [60,61]. The range of research on NO free radicals as messengers involved in plant immune responses has increased gradually [62,63]. Under the stimulation of pathogens, the NO synthesis efficiency increases, which can induce the accumulation of phytoalexins, affect the accumulation of peroxidase ROS, induce the generation of H_2_O_2_, regulate the redox state of the host, and activate the expression of MAPK defense genes and disease-related proteins [64]. Many studies have shown that NO regulates the HR–PCD and activates the expression of plant disease resistance- and defense-related genes through synergistic effects with ROS [65]. During the compatible interaction between tobacco and the necrotrophic fungus *Botrytis cinerea*, both NO and ROS increase, and SA-induced protein kinases and some defense related genes are activated [66]. A loss of function and virus-induced gene silencing analyses have shown that NO plays key roles in the resistance to *B. cinerea* and in inducing *PR-1* expression. In contrast, ROS are positively correlated with lesion expansion [67,68]. In the HR reaction, NO can also interact with SA to regulate the production of ET, and finally affect the formation of an HR [69]. After plants perceive a pathogen infection, an oxidative burst promotes the accumulation of NO and ROS and activates the downstream signal cascade. However, the production and transmission of NO in plants, and the regulation of downstream genes, are still vague. In the future, the untapped role of NO in gene transcription should be further explored using omics technology.

## 3. Plant Defense Hormone-Mediated Signaling Pathways

### 3.1. SA-Mediated Disease Resistance Signaling Pathway

**Figure 3:**
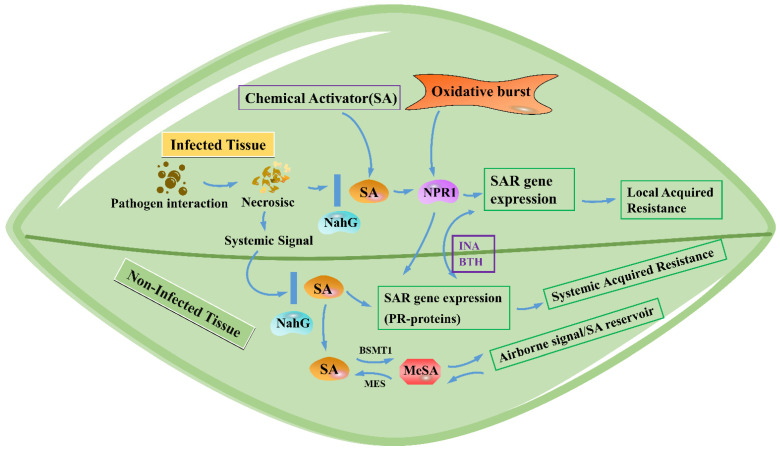
The function of SA in the formation of local resistance and SAR. Plants can accumulate a large amount of SA after being infected by many pathogens. In addition to the SA accumulated at the infection site, SA is also associated with the SAR of uninfected tissues. NPR1 is critical for SA-dependent PR gene expression and SAR. The inhibition of SA accumulation or biosynthesis will inhibit the formation of SAR. NahG is an inhibitor of SA synthesis, which can convert SA into inactive catechol. Therefore, the overexpression of this gene can inhibit SAR formation and PR gene expressions. INA and BTH are analogues of SA, which are also plant defense activators and can induce SAR and the expression of PR genes. MeSA is a derivative of SA, which can act as a mobile inducer of SAR and induce the expression of defense genes in adjacent plants. SAR, systemic acquired resistance; MeSA, methyl salicylate; BTH, benzothiadiazole; INA, 2,6-dichloroisonicotinic acid; NahG, salicylate hydroxylase; BSMT1, benzoic acid/salicylic acid carboxyl methyltransferases; MES, methylesterases.

In recent years, research on the function of SA has become an important and rapidly developing field in biology. SA plays a wide range of physiological roles in plant growth, development, maturation, aging regulation, and stress-resistance induction. However, the current research on the physiological roles of SA in plants is still focused on disease resistance and signal transduction [70,71,72]. After infection, SA is closely related to the formation of the local resistance of infected tissues and the SAR of uninfected tissues (Figure 3). Infected tissues show the HR response, produce signaling substances such as SA, and activate SAR gene expression [73,74]. SA, as a system signal, can also cause an increase in the SA level in uninfected tissues and then induce the expression of *PR* genes, resulting in disease resistance throughout the whole plant [75].

Plant SAR is realized through the mutual recognition and interaction between plant resistance genes (*R*) and the avirulence genes of pathogenic microorganisms [76]. SA is a signal molecule of plant systemic resistance responses that is specific to many *R* genes, and which can regulate many immune-related genes [77] (Shine et al. 2016). The SA accumulation caused by pathogen infection can be controlled by some protein regulators, such as enhanced disease susceptibility 1 (EDS1), phytoalexin-deficient 4 (PAD4), EDS4, EDS5, and non-race-specific disease resistance 1 (NDR1) [78,79,80,81]. EDS1 and its interaction factors, including PAD4 and the sensitivity-associated senescence-associated gene 101 (SAG101), are involved in regulating the accumulation of HR–PCD and SA. In turn, SA can enhance the expression of *EDS1*/*PAD4*/*SAG101* through a positive feedback loop [82,83,84]. Moreover, EDS1 and SA have redundant functions in some coiled-coil nucleotide-binding site–leucine-rich repeat (CC-NB-LRR) protein-mediated signal transduction [85]. NDR1 is an R protein containing a CC domain located in the plasma membrane. The *Arabidopsis NDR1* gene is a positive regulator of SA accumulation and is necessary for *R* gene-mediated signaling. As with *EDS1*, *NDR1* acts upstream of SA to regulate SA accumulation, and the induction of the SA/*NDR1*-mediated pathway may increase plant resistance to pathogens [81,86].

SA is perceived by two classes of receptors: transcriptional activator non-expressor of *PR* genes 1 (NPR1) and the transcriptional repressors NPR3/NPR4. NPR1’s encoding of a redox-sensitive protein results in an important regulatory element in the SA signaling pathway. NPR1 is a key component of SA-induced SAR [87]. When there is no pathogen infection, NPR1 is continuously cleared by proteasomes to limit the activity of its coactivator, thereby preventing untimely SAR activation. However, not all SAR-mediated responses depend on NPR1. The *Arabidopsis* whirly transcription factor (AtWHY1) and SSI1 are two regulators of SAR that depend on SA and not on NPR1 [88,89,90]. The activation of NPR1 also regulates SA tolerance, the expression of isochorismate synthase 1 (*ICS1*), and SA accumulation in *Arabidopsis* [86]. As a transcription inhibitor, NPR3/NPR4 inhibits the response of the SA pathway in the absence of pathogen infection, which is the opposite function of NPR1 in plant immune regulation. After binding with SA, its transcriptional repressor activity is inhibited, activating downstream target genes and defense responses [91]. Recent studies have shown that SAR requires the opposite effects of NPR1 and NPR3/NPR4, which may also contribute to the production of PTI and ETI [92].

### 3.2. JA-Mediated Disease Resistance Signaling Pathway

JA is also a stress signal molecule that accumulates rapidly and massively when plant tissues are invaded by microbial pathogens or insects [93]. JAs may induce the expression of defense-related proteins, such as polyphenol oxidase, protease inhibitors, peroxidase, and lipoxygenase. JAs may also induce the production of alkaloids and some volatiles, and the formation of defense structures, which exert the stress and disease resistance functions of plants [94].

Although there is a good understanding of the JA synthesis pathway, the perception and subsequent signal transduction of JA are not very clear. The jasmonate ZIM domain (JAZ) is known to be an inhibitor of the JA signaling pathway that inhibits the expression of JA response genes through interactions with MYC2. JA signals can promote the interaction between JAZ and SCF^COI1^ ubiquitin ligase, resulting in the ubiquitination of the JAZ protein and degradation by the 26S proteasome [95]. After JAZ protein degradation, transcription factors such as MYC2 are promoted to activate JA response genes [96,97,98]. Recent studies have shown that JAZ4, a family member of JAZ, enhances resistance to the bacterial pathogen *P. syringae* pv. *tomato* strain DC3000 by inhibiting the JA pathway [99]. Phytochrome and flowering time 1, which encodes the subunit of the mediator complex, is a key regulator of JA-dependent defense responses in *Arabidopsis*, and it presumably acts downstream of the COI1–JAZ–MYC2 complex. In combination with another mediator complex subunit, MED8, it is involved in regulating plant growth and development (such as flowering time) and JA-dependent defense responses [100]. There may be crosstalk between the JA signaling pathway and antiviral RNA-silencing pathways. The JA-responsive transcription factor JAMYB transcriptionally activates *Argonaute18*, the core component of RNA silencing, to increase the resistance of rice to viral diseases [101]. In addition, JA suppresses the brassinosteroid (BR) pathway in a COI1-dependent manner in response to rice black-streaked dwarf virus infection [102]. However, BR and JA positively regulate rice stripe virus resistance in the rice–virus interaction [103].

### 3.3. ET-Mediated Disease Resistance Signaling Pathway

ET plays an important regulatory role in many physiological processes of plants, from seed germination to senescence [104]. ET biosynthesis is regulated by many factors, including developmental and environmental factors [105]. There are five ET membrane-binding receptors in *Arabidopsis*, ETR1, ETR2, ERS1, ERS2, and EIN4, which can transmit signals to downstream effectors. Arabidopsis infected by *Fusarium oxysporum* can stimulate ETR1-mediated ET signaling and enhance susceptibility. Additionally, ETR1 receptor mutant plants can significantly increase the resistance compared with wild-type plants, but other ET mutants, including *ein2*, *ein5*, and *ein4*, are as susceptible as wild-type plants [106]. CTR1 is a serine/threonine protein kinase belonging to the Raf family that is located downstream of the ET receptor. In the absence of an ET signal, the ET receptor activates CTR1, which negatively regulates the downstream ET response pathway [107]. After binding to ET, the receptor is inactivated, resulting in the inactivation of CTR1, and EIN2 becomes a positive regulator of the ET pathway [108]. EIN2 transmits a signal to the EIN3 transcription factor located in the nucleus, causing EIN3 to bind to the ET response element in the promoter of ET response factor 1 (ERF1). ERF1 can interact with the GCC box of the target gene promoter to activate the downstream ET response [109]. The ineffective mutation of EIN2 leads to insensitivity to ET during the whole plant developmental process, indicating that EIN2 is a key positive regulator in the ET signal transduction pathway. EIN3 is a member of a multigene family of nuclear localization proteins in *Arabidopsis*. Among the six members of this family, *EIN3*, *EIN3-like 1 (EIL1*), and *EIL2* can restore the phenotypes of *EIN3* mutants, indicating that *EIL1* and *EIL2* are also involved in ET signal transduction [110,111].

JA and ET jointly mediate rhizosphere microorganism-triggered induced systemic resistance (ISR), which can improve the resistance of plants to a broad spectrum of pathogens, such as *Alternaria brassicicola* [112], *Botrytis cinerea* [113], and *Pseudomonas syringae* pv. *tomato* DC3000 [114]. *ERF1* and *MYC2* integrate signals from the JA/ET signal transduction pathway and activate defense-related genes, such as *PR-3*, *PR-4*, and *PR-12*, which encode antimicrobial peptides involved in the JA/ET response [115]. Further studies showed that the expression of *PDF1.2* depends on the simultaneous activation of JA and ET signals, whereas the expression of *Thi2.1* only depends on methyl jasmonate (Me-JA) [116,117]. In addition, ET can also produce inducible systemic resistance in the *jar1* mutant, indicating that the elements of the ET response act downstream of JA in the signal transduction process of inducible systemic resistance [118].

## 4. Heterotrimeric G Protein-Mediated Disease Resistance Signaling Pathway

The growth, development, and differentiation of plant cells are controlled by the stimulation of plant internal factors and the external environment. A considerable number of regulatory factors do not enter the cell, but are transformed into intracellular signals through transmembrane signaling pathways, in which the signal transduction components on the plasma membrane play crucial roles [119]. Among them, heterotrimeric G proteins are currently considered to be ubiquitous signal transduction elements in eukaryotes, including plants, fungi, and animals [120]. The heterotrimeric G protein signaling pathway not only converts extracellular signals into intracellular signals, but it also functions to amplify signals and activate multiple downstream effector enzymes, such as adenylate cyclase in animals and phospholipase C (PLC) in plants [121,122]. Effector enzymes can produce a large number of second messengers, such as cAMP, which can also further amplify signals through signal transduction pathways [123].

In *Arabidopsis* and rice, the typical heterotrimeric G protein consists of one α subunit, one β subunit, and two γ subunits. Heterotrimeric G protein signaling in plants is related to a variety of plant responses to biotic/abiotic stresses and the regulation of plant physiological growth and development [124]. In particular, heterotrimeric G proteins are involved in the resistance and immunity of plants to a variety of pathogens [125,126]. Arabidopsis G proteins could directly interact with the FLS2–BIK1 receptor complex to modulate flg22-triggered immunity through both pre-activation and post-activation mechanisms [127,128]. In *Arabidopsis*, resistance to the necrotrophic pathogenic fungi *A. brassicola* and *F. oxysporum* depends on the Gβγ-mediated signaling pathway, and interfering with the Gβγ signaling pathways later affected many JA-mediated responses. Therefore, it has been speculated that G proteins may participate in plant resistance to necrotrophic pathogenic fungi by enhancing the JA signaling pathway [129]. The heterotrimeric G protein β subunit is necessary for plant resistance against different *P. syringae* strains. It activates MAPK signaling and produces ROS through NADPH oxidase downstream of the G protein signal [130]. The MAPK cascade can be used as a downstream effector of G protein signal transduction. RACK1 is a plant MAPK protein connecting heterotrimeric G proteins and the MAPK cascade to form a unique signal pathway in plant immunity [131]. However, the mechanisms behind the ability of G protein complexes to trigger the activation of the MAPK cascade are still unknown. Moreover, how the upstream and downstream components of heterotrimeric G protein signaling participate and how the G protein regulates their active metabolism need to be explored.

## 5. MAPK Cascade-Mediated Disease Resistance Signaling Pathway

Protein kinases are the target enzymes of many intracellular second messengers, which further transduce signals by regulating the phosphorylation of intracellular proteins [132]. In plants, there are more than 30 kinds of known protein kinases, and they affect many processes, including light resistance, cold resistance, photosynthesis, self-incompatibility, and cell division [133,134]. Among the protein kinases, MAPK is a type of Ser/Thr protein kinase, and it has three main types: MAPKs, MAPKKs, and MAPKKKs [40]. They form a series of reactions that transfer and magnify external signals. MAPKKKs are located upstream of the chain reaction and link external signals with downstream regulators by phosphorylation [135]. An activated MAPKKK activates MAPKK, and then the MAPK is activated through dual phosphorescence by MAPKK at Thr/Tyr phosphorylation sites. MAPK stays in the cell to activate a series of other protein kinases or enters the nucleus to regulate the expression of certain genes through transcription factor phosphorylation [136]. MAPK signaling actively participates in PTI and ETI responses and mediates a variety of defense mechanisms in response to pathogen infection [137,138,139]. *Arabidopsis MKK7* belongs to group D of plant MAPKKs. Group D members from other species also play key roles in plant defense responses. For example, the overexpression of *MKK4* from tomato leaves leads to cell death and activates *MPK2* and *MPK3*, both of which are related to plant defense responses [140]. The overexpression of *MKK7* in *Arabidopsis* can positively regulate basic resistance and SAR by regulating SA synthesis. This is characterized by constitutive *PR* gene expression and enhanced resistance to pathogens [141]. The transcriptional activation of *MPKs* depends on their phosphorylation. Under pathogen attack, cotton *GhNTF6* is the only member of the MPK family that requires a phosphorylation reaction, and the overexpression of this gene in *Arabidopsis* enhances resistance against *Verticillium dahlia* [142]. WRKY is a downstream transcription factor in the MAPK pathway, and its resistance role has been reported in many plants. An RNA-Seq analysis of rice WRKY67 shows that it can induce the transcription of defense-related genes, and the overexpression of *WRKY67* in rice can increase resistance to bacterial blight [143].

The reverse phosphorylation of protein phosphatase is a termination signal or a reverse regulation. Although research on protein phosphatase is not as in-depth as that on phosphokinases, it is of equal significance. The results of protein phosphorylation and dephosphorylation are different, and this may be related to the transduction of different stimulating signals in cells. In fact, it is the reversibility of protein phosphorylation that provides a switching effect for cell information that enables cells to effectively and economically regulate their responses to internal and external information.

## 6. ncRNA-Mediated Disease Resistance Pathway

Non-coding RNAs (ncRNAs) refer to RNAs that do not encode proteins, which play an essential role in plant growth and development and resistance to abiotic/biotic stresses. ncRNAs involved in the regulation of plant innate immunity mainly include microRNAs (miRNAs), long non-coding RNAs (lncRNAs), and circular RNAs (circRNAs), which play an important role in plant immune responses [144,145]. As short-chain (21–25 nt) ncRNAs, miRNAs usually regulate gene expression by cleaving or inhibiting the translation of target gene transcripts, and play critical roles in regulating plant development and immune responses [146,147]. In *Arabidopsis*, tobacco, barley, and other species, miRNAs can regulate *R* gene expression by guiding the cleavage of *R* genes or triggering phasiRNA production, indicating that miRNAs play a role in regulating plant PTI or ETI [148,149,150]. Moreover, some miRNAs and small interfering RNAs (siRNAs) can directly target key signaling components such as immune receptors, receptor-like kinases, or downstream transcription factors to modulate immunity [151,152]. Disease resistance and high yields in plants are often antagonistic. miR393 can enhance plant immunity by fine-tuning the balance of SA–auxin hormone and promoting the exocytosis of antibacterial proteins in plants [153]. In rice, miR396 and its target *growth-regulating factor* (*OsGRF*) genes modulate the trade-offs between rice blast disease resistance and yield [154]. However, the specific mechanisms by which most miRNAs regulate disease resistance are still poorly understood.

ncRNAs with a length greater than 200 nt are called lncRNAs, and some lncRNAs serve as precursors of miRNAs and siRNAs to assist in the cleavage of target genes [155,156]. lncRNAs can regulate ROS accumulation and induce the expression of *PR* genes to modulate plant immunity [157,158]. In tomato, lncRNA16397 induces the expression of *glutaredoxin* (*GRX*) to reduce the accumulation of ROS in cells, thereby reducing the damage to the cell membrane and enhancing the resistance to *Phytophthora infestans* [159]. In addition, some lncRNAs serve as targets of miRNAs or endogenous target mimics for miRNAs to regulate host immunity [157,160]. Circular RNA (circRNA) is another type of lncRNA, which is a closed-loop single-stranded RNA formed by the reverse splicing of introns or gene exons and delivered to the cytoplasm. Plant circRNAs can directly or indirectly bind to miRNA to competitively inhibit the regulation of miRNA on target genes, and are widely involved in the regulation of plant immune responses [145,161].

Many studies have revealed that mobile ncRNAs, mainly miRNAs, can translocate between plants and pathogens to mediate cross-kingdom RNAi [162,163]. Notably, virus-derived small interference RNAs (vsiRNAs) were shown to be a potential trigger for virus resistance via RNA silencing-related antiviral mechanisms or via regulating host defensive gene expression in transgenic plants [164,165]. Thus, ncRNA-mediated regulation plays an important role in plant disease resistance, but the regulatory mechanisms and roles of ncRNAs in immunity response need to be further studied.

## 7. Interaction and Complexity of Disease Resistance Signaling Pathways

In nature, plants often need to fight simultaneously against a variety of pathogens, and this alters the defense responses originally induced by plants. Therefore, plants need effective regulatory mechanisms to adapt to the changes in a hostile environment. The crosstalk between signaling pathways provides the plant with a powerful capacity to finely regulate defense responses [166]. Plants can activate an effective defense response by regulating the level of signal molecules, changing the expression of defense-related genes, and coordinating the complex relationships among defense signaling pathways.

There are at least two signaling pathways against different pathogens in *Arabidopsis*, the SA-dependent and JA/ET-mediated pathways. Many studies have shown that there is crosstalk between SA- and JA/ET-induced signaling pathways, and most evidence shows that the SA and JA/ET pathways have an antagonistic relationship (Figure 2) [167,168,169,170]. Perhaps because of this, the activation of SA responses may make plants more vulnerable to attackers connected to the JA/ET defense response [171]. Robert showed that the antagonistic effect of SA on the JA signal transduction pathway is likely to be achieved by inhibiting the JA-mediated degradation of the JAZ protein [172]. However, some studies have shown that there is a synergistic relationship between the SA and JA signaling pathways [173]. JA and ET co-regulate and activate the expression of some defense-related genes, such as *PR-3*, *PR-4*, and *PR-12* [174]. ERF1 is a positive regulator of JA and ET signal transduction. Recent studies have found that the overexpression of rice *JAZ1* significantly inhibited the transcriptional expression of *ERF1*, confirming the synergy between the JA and ET pathways [175]. However, JA and ET do not always cooperate with each other. For example, the wound response gene activated by JA is inhibited by ET, and the activation of JA by the transcription factor *MYC* inhibits their respective transcriptional activities by binding with an ET transcription factor, resulting in the weakening of plant defenses against insect attacks [176].

The interaction between plants and pathogens is generally accompanied by intracellular Ca^2+^ transients, which activate the Ca^2+^ signaling pathway, induce the production of ROS and NO, and positively regulate early local and systemic acquired resistance (Figure 2) [177]. SA and ROS also have a common disease resistance signal-sensing mechanism. Recent studies have shown that small defense-associated protein 1 can regulate plant immunity through the SA-mediated defense pathway and induce a tolerance to oxidative stress [178]. However, the relationship between SA-mediated defense responses and the Ca^2+^ signaling pathway is unclear. SA and ROS also have a common molecular signal-sensing mechanism of disease resistance. In wheat spotted-leaf mutants, the SA accumulation and enhanced Ca^2+^ signaling jointly triggered PCD, which eventually led to spontaneous leaf necrosis [179]. However, it is not clear whether or how SA is regulated by the Ca^2+^ signal transduction pathway during plant–pathogen interactions.

Regulatory proteins are the basis of crosstalk between different defense signal pathways, and they are also the key factors regulating the overall disease resistance of plants. At present, some key regulatory proteins involved in the crosstalk between the SA and JA/ET pathways and the coordination of their complex relationships, such as NPR1, EDS1 and MPK4, have been identified in *Arabidopsis* by gene mutation and gene transgenic techniques (Table 1), and this enables us to better understand the interaction mechanisms among different signaling pathways. However, although progress has been made in the study of plant disease resistance signaling pathways, the locations and mechanisms of synergistic or antagonistic interactions among pathways still lack direct experimental evidence. In addition, how plants coordinate these complex relationships and the corresponding molecular mechanisms need to be further studied.

## 8. Conclusions and Prospects

Plant diseases have always been a considerable problem that limit the achievement of high and stable yields, high-quality produce, and the safe production of crops. In-depth studies and the utilization of plant disease resistance mechanisms and disease resistance-related signaling pathways will provide new ideas for the prevention and control of plant diseases. The mechanisms of plant disease resistance responses are very complex and diverse. There are huge numbers of regulatory factors and genes involved in plant–pathogen interactions, disease resistance signal transduction, and defense responses, and they form a complex regulatory network (Figure 2). Signaling molecules, such as Ca^2+^, H_2_O_2_, NO, SA, JA, ET, and the heterotrimeric G protein, play important roles in this network. At present, studies have shown that other signaling molecules, such as abscisic acid, gibberellic acid, cytokinin, BR, and peptide hormones, are also involved in plant defense-related signal transduction pathways, but their roles in plant defense are still unclear.

Plant hormone regulation involves complex signal transduction networks, such as those involved in growth, development, and environmental stress responses. At present, with the development of modern molecular biology and transgenic technology, substantial progress has been made in the identification of the key components in plant signal transduction and in the understanding of plant hormone signal transduction (especially for SA, JA, and ET). Now, an important issue is how different hormone-mediated developmental processes and defense responses are regulated in specific tissues and cells. In addition, not only can plants produce hormones, but some plant pathogens can also produce plant hormones, which can cause hormone imbalances and break the plant defense response system. It is not clear how pathogens regulate hormone signal transduction elements to make plants susceptible. An in-depth understanding of hormone-mediated defense responses is very important for controlling crop diseases and insect pests.

Plant disease resistance signaling pathways are not isolated, but they are inter-connected through complex regulatory networks. Understanding how plants coordinate various signal components is an important aspect of future research. In recent years, many researchers at home and abroad have tried to identify the regulatory proteins acting between signaling pathways, but the results have not been consistent, and their specific functions and roles in hormone signaling pathways need to be studied further. Finding new contact points will be one of the hotspots in the research on plant disease resistance signaling pathways in the future. With the development of molecular biology and the in-depth study of disease resistance-related transcription profiles from some model plants, such as *Arabidopsis* and rice, many plant regulatory protein-encoding genes related to pathogen infection have been extensively studied and analyzed. Using plant genetic engineering technology, various transgenic disease-resistant plants have been constructed and applied in agricultural production. This may increase our understanding of plant resistance mechanisms and also enrich the candidate genes for crop disease resistance breeding and improvement, which has particularly important research significance and broad application prospects.

## Figures and Tables

**Table 1:** Key regulators in SA and JA/ET crosstalk.

Gene Name	Species	Biological Function	Molecular Switch Function	Citations
*NPR1*	*A. thaliana*	SA-dependent PR gene expression and SAR	SA signaling (+) JA/ET signaling (−)	[180]
*EDS1*	*A. thaliana*	*R*-mediated resistance; regulation of HR/PCD and SA accumulation	SA signaling (+) JA/ET signaling (−)	[80,82,181]
*PAD4*	*A. thaliana*	EDS1 interactor, basal and receptor-triggered immunity, and SA accumulation	SA signaling (+) JA/ET signaling (−)	[84,182,183]
*WRKY70*	*A. thaliana*	Regulator of developmental senescence and plant defense	SA signaling (+) JA/ET signaling (−)	[184,185]
*WRKY52*	*Vitis quinquangularis*	SA-dependent signaling pathway and in HR cell death	SA signaling (+) JA/ET signaling (−)	[186]
*WRKY62*	*A. thaliana*	Downstream of cytosolic NPR1	SA signaling (+) JA/ET signaling (−)	[167,187]
*WRKY13*	*O. sativa*	Enhances rice resistance to bacterial blight and fungal blast	SA signaling (+) JA/ET signaling (−)	[188]
*GRX480*	*A. thaliana*	TGA-interacting protein, controlling the redox state of regulatory proteins	SA signaling (+) JA/ET signaling (−)	[189]
*MPK4*	*A. thaliana*	JA-responsive gene expression; represses SAR	SA signaling (−) JA/ET signaling (+)	[182,190]
*SSI2*	*A. thaliana*	Regulates the overall level of desaturated FAs	SA signaling (−) JA/ET signaling (+)	[191]
*WRKY33*	*A. thaliana*	Defense toward necrotrophic fungi	SA signaling (−) JA/ET signaling (+)	[192]
*RST1*	*A. thaliana*	Lipid synthesis; cuticular wax with embryo development	SA signaling (+) JA/ET signaling (−)	[193]

+ positive regulator of the pathway; − negative regulator of the pathway.

## Data Availability

Not applicable.
